# Morphological aspects and Cox-2 expression after exposure to 780-nm laser therapy in injured skeletal muscle: an *in vivo* study

**DOI:** 10.1590/bjpt-rbf.2014.0057

**Published:** 2014

**Authors:** Natalia C. Rodrigues, Roberta Brunelli, Daniela C. C. Abreu, Kelly Fernandes, Nivaldo A. Parizotto, Ana C. M. Renno

**Affiliations:** 1 Departamento de Fisioterapia, Universidade Federal de São Carlos (UFSCar), São Carlos, SP, Brazil; 2 Departamento de Cirurgia, Faculdade de Ciências Médicas, Universidade Estadual de Campinas (UNICAMP), Campinas, SP, Brazil; 3 Departamento de Biomecânica, Medicina e Reabilitação do Sistema Locomotor, Universidade de São Paulo (USP), Ribeirão Preto, SP, Brazil; 4 Departamento de Biociências, Universidade Federal de São Paulo (UNIFESP), Santos, SP, Brazil

**Keywords:** collagen, cryolesion, cyclooxygenase 2, inflammation process, low-level laser therapy, physical therapy

## Abstract

**Background:**

The effectiveness of low-level laser therapy in muscle regeneration is still not well known. Objective: To investigate the effects of laser irradiation during muscle healing.

**Method:**

For this purpose, 63 rats were distributed to 3 groups: non-irradiated control group (CG); group irradiated at 10 J/cm² (G10); and group irradiated at 50 J/cm² (G50). Each group was divided into 3 different subgroups (n=7), and on days 7, 14 and 21 post-injury the rats were sacrificed.

**Results:**

Seven days post-surgery, the CG showed destroyed zones and extensive myofibrillar degeneration. For both treated groups, the necrosis area was smaller compared to the CG. On day 14 post-injury, treated groups demonstrated better tissue organization, with newly formed muscle fibers compared to the CG. On the 21st day, the irradiated groups showed similar patterns of tissue repair, with improved muscle structure at the site of the injury, resembling uninjured muscle tissue organization. Regarding collagen deposition, the G10 showed an increase in collagen synthesis. In the last period evaluated, both treated groups showed statistically higher values in comparison with the CG. Furthermore, laser irradiation at 10 J/cm2 produced a down-regulation of cyclooxygenase 2 (Cox-2) immunoexpression on day 7 post-injury. Moreover, Cox-2 immunoexpression was decreased in both treated groups on day 14.

**Conclusions:**

Laser therapy at both fluencies stimulated muscle repair through the formation of new muscle fiber, increase in collagen synthesis, and down-regulation of Cox-2 expression.

## Introduction

Innovative clinical approaches to accelerate muscle metabolism and to repair damage to muscle tissue are being developed, including low-intensity pulsed ultrasound (LIPUS), pulsed electric magnetic field, and extracorporeal shock wave therapy^1^. Low-level laser therapy (LLLT) is also a common modality used to treat skeletal muscle conditions^2^^-^ 4, and several studies have demonstrated that it is effective in reducing post-injury inflammatory processes, accelerating soft tissue healing, and stimulating the formation of new blood vessels^5^^,^ 6. Furthermore, there is evidence that LLLT has positive effects on muscle tissue and muscle repair^2^^,^ 3. It seems that laser therapy is able to increase myoblast motility and accelerate muscle fiber formation^5^. Also, Baptista et al.2 demonstrated that 660 nm laser at 5 J/cm2 promoted an increase in collagen IV immunolabeling in skeletal muscle 7 days after injury. This result corroborates Renno et al.3, who also found that laser therapy was capable of decreasing the necrosis area and producing better tissue organization at the site of the injured muscle. Recent studies have shown that LLLT can improve muscle regeneration through many biochemical process modulations, including the production of pro- and anti-inflammatory cytokines such as cyclooxygenase enzyme^7^, increased collagen synthesis, maintenance of the functional integrity of muscle fibers^7^, enhanced mitochondrial respiration, and adenosine triphosphate (ATP) formation4. Despite the positive effects of LLLT on tissue regeneration, the mechanism by which this therapy acts on muscle tissues is not fully understood and, for many, its use as a treatment modality is still controversial^8^. Moreover, in the literature different authors use a wide range of wavelengths and fluencies in the treatment of muscle injury, which makes it difficult to compare published results and determine an ideal treatment protocol. Almeida et al.^9^ suggest that laser radiation at infrared wavelength can penetrate better through human skin than red wavelength. This possibility was confirmed by Brunelli et al.^10^, who found positive results, such as more blood vessels, better organization of muscle fibers, high MyoD levels, and reduction of lesion area, using 780 nm LLLT with two different fluencies of 10 and 50 J/cm^2^. 

In this context and in an attempt to determine the best parameters, the aim of this study was to verify the effects of the LLLT parameters used by Brunelli et al.^10^, i.e. infrared 780 nm laser therapy at two different fluencies (10 and 50 J/cm^2)^, on injured skeletal muscle by means of the histopathological, collagen, and Cox-2 immunoexpression analyses of aspects of structural support and the inflammatory process.

## Method

### Animals

Sixty-three Wistar male rats (weighing 300±20 g) were used in the current study. They were kept under controlled temperature (22+2ºC), light-dark periods of 12 hours, with free access to water and commercial diet. All animal handling and surgical procedures were conducted strictly according to the Guiding Principles for the Care and Use of Laboratory Animals. This study was approved by the Ethics Committee of Universidade Federal de São Carlos (UFSCar), São Carlos, SP, Brazil (protocol no. 068/2009). The animals were randomly distributed to 3 groups: non-irradiated control group (CG); group irradiated at 10 J/cm² (G10); and group irradiated at 50 J/cm² (G50). Each group was divided into 3 different subgroups (n=7) and on days 7, 14 and 21 post-injury, the rats were sacrificed. 

### Experimental design

### Surgery

The animals were subjected to anesthesia with Xilazin (Syntec(r), 20 mg/kg, IP) and Ketamin (Agener(r), at 40 mg/kg, IP) and exposed to cryolesion of the right tibialis anterior (TA) muscle. The cryolesion consisted of two freeze-thaw cycles of the muscle in situ. Freezing was carried out by applying the flat top end (0.5×0.5 cm) of a piece of iron, precooled in liquid nitrogen, to the surface on the middle belly of the muscle. This position was sustained for 10s (twice)11. Once the muscles had thawed, the wounds were closed with polyamide threads (6-0), and thereafter, the animals were kept for several hours on a warm plate (37 ºC) to prevent hypothermia.

### Laser irradiation

A low-energy Ga-Al-As laser (MM Optics, São Carlos, SP, Brazil), 780 nm continuous wavelength, 4.0 mm2 beam diameter, with 10 J/cm² (20 mW; 20 seconds of irradiation; total energy per point 0.4 J) and 50 J/cm² (40 mW; 50 seconds of irradiation; total energy per point 2 J), was used in this study. The irradiation was applied to one point above the area of the injury through the punctual contact technique. The treatments started 48 hours post-surgery and were performed 5 times/week (every 24 hours), followed by a 48-hour interval. On 7, 14, and 21 post-injury days, animals were sacrificed by CO2 suffocation in order after their TA muscles had been extracted. Those irradiation regimens were selected to reproduce the same regimens used at the clinic.

### Histopathological analysis

Muscles obtained from all experimental and control groups were washed immediately with saline and then fixed in 10% buffered neutral formalin solution. After fixation, the muscle tissue was embedded in paraffin. Then, five histological serial sections (5 μm) were obtained from the middle belly of each TA muscle and stained with hematoxylin and eosin (H&E stain, Merck). Histopathological evaluation was performed by an experienced pathologist (DAR) who was blinded to the treatment using a light microscope (Olympus Optical Co. Ltd., Tokyo, Japan) at 40x magnification. The qualitative analysis considered changes to the site of the injury, such as the presence of inflammatory processes, granulation tissue, necrosis area, focal or diffuse myofibrillar degeneration. Tissues undergoing hyperplastic, metaplastic and/or dysplastic changes were also investigated in each animal12.

### Collagen analysis

The amount of collagen at the site of the injury was measured through the picrosirius-polarization method13. The histological sections were stained with picrosirius red, which allows the assessment of structural changes to the new muscle fibers under polarized light. This method also allows the quantitative evaluation of the stage of muscle organization based on the birefringence of the collagen fiber bundles after staining with picrosirius and hematoxylin.

### Immunohistochemistry

Serial longitudinal muscle sections (4 μm) were deparaffinized in xylene and rehydrated in graded ethanol, then pretreated by microwave (Brastemp, SP, Brazil) with 10 mM citric acid buffer (pH=6) for 3 cycles of 5 min each at 850 W for antigen retrieval. The material was pre-incubated with 0.3% hydrogen peroxide in PBS for 5 min for inactivation of endogenous peroxidase, and then blocked with 5% normal goat serum in PBS solution for 10 min.

The specimens were then incubated with anti-Cox-2 (Santa Cruz Biotechnology, USA) at a concentration of 1:400. Incubation was carried out overnight at 4 ºC in the refrigerator and followed by two washes in PBS for 10 min. The sections were then incubated with biotin-conjugated secondary antibody (anti-rabbit immunoglobulin G - IgG) (Vector Laboratories, Burlingame, CA, USA) at a concentration of 1:200 in PBS for 1 hour. The sections were washed twice with PBS before the application of preformed avidin biotin complex conjugated to peroxidase (Vector Laboratories, Burlingame, CA, USA) for 45 min. The bound complexes were visualized by the application of a 0.05% solution of 3-3'-diaminobenzidine and counterstained with Harris hematoxylin. For control studies of antibodies, the serial sections were treated with rabbit IgG (Vector Laboratories, Burlingame, CA, USA) at a concentration of 1:200 in place of the primary antibody. Additionally, internal positive controls were performed with each staining bath. 

Cox-2 immunoexpression was evaluated both qualitatively (presence of the immunomarkers) and quantitatively. Both analyses were performed in five predetermined fields using a light microscope (Leica Microsystems AG, Wetzlar, Germany) according to a previously described scoring scale from 1 to 4 (1=absent, 2=weak, 3=moderate, and 4=intense) for immunohistochemical analysis13-15. The analysis was performed by two observers (NCR and CT) in a blinded fashion.

### Statistical analysis

Data from the picrosirius analysis were evaluated using two-way ANOVA, followed by the posthoc Newman-Keuls test. The level of statistical significance was defined as p<0.05, with statistical power of 95%. Statistical evaluation was carried out using GraphPad Prism 4 (GraphPad Software, San Diego CA, USA).

## Results

### Histopathological findings

**Figure 1:**
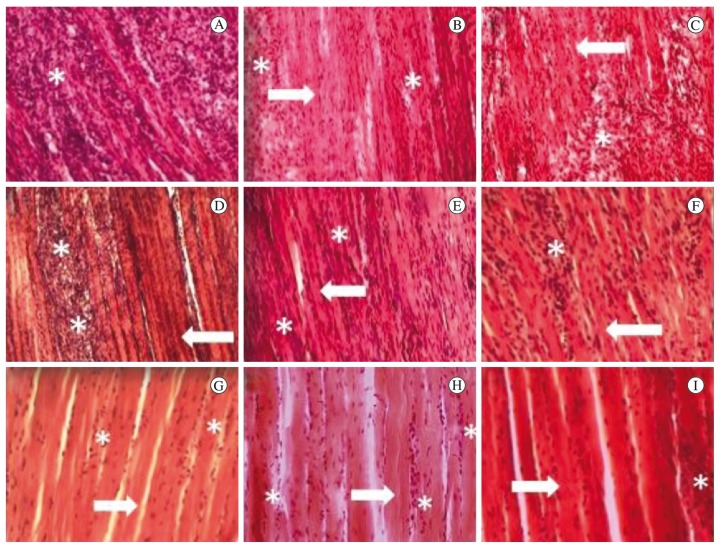
Injured muscle 7 days after surgery: (A) control group; (B) animals irradiated at 10 J/cm2; (C) animals irradiated at 50 J/cm2. Injured muscle 14 days after surgery: (D) control group; (E) animals irradiated at 10 J/cm2; (F) animals irradiated at 50 J/cm2. Injured muscle 21 days after surgery: (G) control group; (H) animals irradiated at 10 J/cm2; (I) animals irradiated at 50 J/cm2. Asterisks indicate granulation tissue. Arrows indicating new fiber muscle. H&E stain. 10 X.

Seven days post-surgery, histopathological findings showed destroyed zones with the presence of significant multifocal cell recruitment, characterizing acute extensive myofibrillar degeneration in the CG (Figure 1-A). In the same way, the G10 presented diffuse cell recruitment and a smaller necrosis area compared to the CG (Figure 1-B). The G50 showed similar morphological findings compared to the CG (Figure 1-C).

On day 14 post-injury, the CG presented destroyed zones and high cell recruitment but less diffuse and more focal when compared to day 7 (Figure 1-D). In the G10, there was lower cell recruitment, an intense presence of newly formed muscle fibers and better tissue organization compared to the CG and G50 (Figure 1-E). Furthermore, the findings of the G10 were similar to those of the G50 (Figure 1-F). On day 21 post-surgery, the histological findings of the CG showed a better structural tissue compared to days 7 and 14 (Figure 1-G). The G10 and G50 showed similar patterns of tissue repair, with an improved muscle structure at the site of the injury and tissue organization resembling that of a non-injured muscle (Figures 1H, 1I).

### Collagen analysis

**Figure 2:**
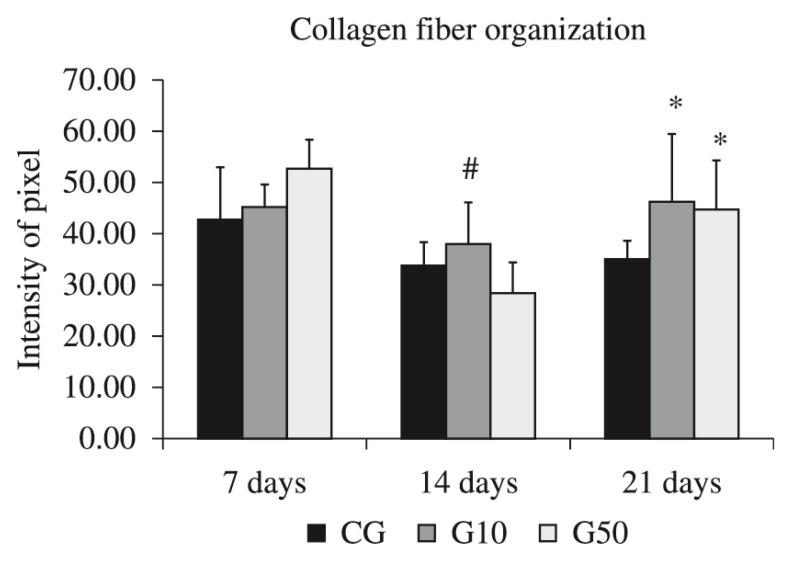
Mean and SD of the collagen evaluation. CG: control group; G10: animals irradiated at 10 J/cm2; G50: animals irradiated at 50 J/cm2. *p<0.05 vs. CG, # p<0.05 vs. G50, according to the Newman-Keuls test.

Seven days post-surgery, picrosirius analysis showed no significant differences among the 3 groups analyzed. Similarly, 14 days after surgery, collagen analysis revealed that there was no significant difference between the CG and G50. However, the G10 (F(1,20), p=0.03) showed statistically higher values when compared to the G50. At 21 days, both treated groups, G10 (F(1,20), p=0.026) and G50 (F(1,20), p=0.019), had statistically higher values compared to the CG (Figure 2).

### Immunohistochemistry

### Cox-2 Immunoexpression

**Figure 3:**
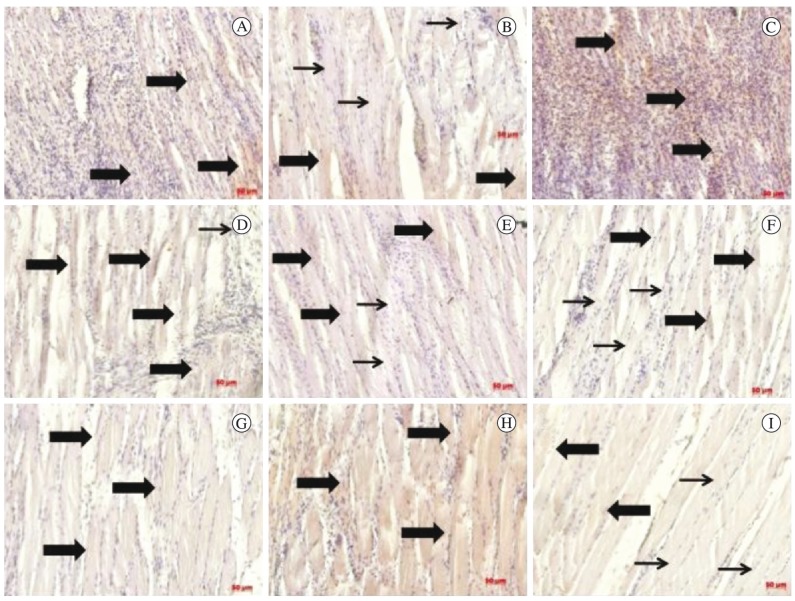
Immunohistochemistry for Cox-2 at 7 days after injury: control group (A); animals irradiated at 10 J/cm2 (B); and animals irradiated at 50 J/cm2 (C). At 14 days after injury: (D) control group; (E) animals irradiated at 10 J/cm2; (F) animals irradiated at 50 J/ cm2. At 21 days after the injury: (G) control group; (H) animals irradiated at 10 J/cm2; (I) animals irradiated at 50 J/cm2. Thick arrows indicate Cox-2 positive cells and thin arrows indicate Cox-2 negative cells. Immunohistochemistry strain. 200X.

Cox-2 expression was consistently observed in the muscle fibers in all experimental groups. Seven days post-surgery, intense Cox-2 immunoexpression was observed in the muscle fibers of the CG (Figure 3-A) and the G50 (Figure 3-C). However, the G10 presented only mild Cox-2 immunoexpression at the site of the injury (Figure 3-B). Fourteen days post-injury, the CG showed higher Cox- 2 immunoexpressivity compared to the other experimental groups, especially at the nucleus of the muscle fibers (Figure 3-D). Interestingly, both treated groups presented moderate expression for this immunomarker (Figures 3-E and 3-F). On day 21 post-injury, a decrease in Cox-2 immunoexpression was observed in the injured muscle fibers of all groups (Figures 3-G, 3-H, and 3-I).

### Quantitative immunohistochemistry analysis

**Figure 4:**
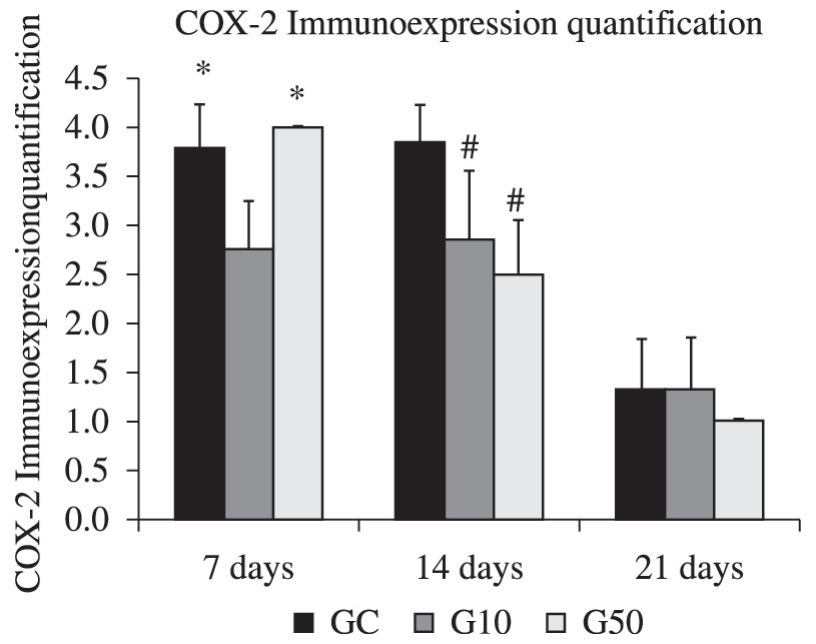
Quantitative evaluation of Cox-2 expression on days 7, 14, and 21 post-surgery. *vs G10; # vs CG and G50.

Figure 4 shows the results of the quantitative analysis of the Cox-2 immunoexpression. Seven days post-surgery, the G10 showed significantly lower Cox- 2 immunoexpression compared to the CG (*F*(7,22), p=0.002) and G50 (F(7,22), p=0.0008). Fourteen days post-surgery, the CG presented significantly higher Cox-2 immunoexpression compared to the treated groups (F(7,22), p=0.002 for G10; p=0.0004 for G50). At this point, similar findings were observed for both laser-irradiated groups. Twenty-one days post-surgery, no difference was observed between the experimental groups (Figure 4).

## Discussion

The aim of this study was to investigate the histomorphological aspects, collagen deposition, and Cox-2 expression after laser irradiation in the different phases of muscle healing. The main results showed that laser irradiated animals showed a decrease in the inflammatory process, decrease in the area of necrosis, and better tissue organization at the site of the injury, especially on days 7 and 14 post-surgery. Moreover, the laser irradiated groups, especially G10, presented an increased amount of collagen and decreased Cox-2 immunoexpression.

The improved histomorphological aspects at the site of the injury, especially in the initial periods of repair, corroborate the results of Silveira et al.^14^, who found that LLLT (904 nm) at 5 J/cm^2^ produced a significant increase in mitochondrial metabolism and in succinate dehydrogenase activities, which may have accelerated the muscle healing process. Also, Cressoni et al.6 found that AlGaInP laser treatment (785 nm) at 12 J/cm^2^ produced a decrease in the number of leukocytes in the inflammatory infiltrate in injured muscles in rats. Rizzi et al.^16^ found that 904 nm laser at 5 J/cm^2^ reduced the inflammatory response induced by trauma and it was capable of blocking the effects of reactive oxygen species (ROS) and the activation of NF-kappaB. Thus, the positive effects of LLLT on modulation of the inflammatory process and stimulation of cell proliferation (such as myogenic precursor cells and fibroblasts) could result in improved tissue healing and better structure at the site of the lesion. 

This study showed that LLLT produced an increase in the amount of collagen at site of injury in the last periods evaluated. Collagen fibers are the main components of the basal lamina and provide structural support for myofibers^17^. They also instruct the satellite cells, myoblasts, and myotubes to migrate, proliferate, and differentiate in muscle fibers^6^. These effects of LLLT on collagen metabolism are supported by other authors, such as Baptista et al.^2^, who found that collagen VI was modulated by LLLT during muscle regeneration, which might be associated with the better tissue outcome. Cressoni et al.^6^ found that LLLT was able to accelerate the formation of connective tissue through the proliferation of fibroblasts and collagen fibers. Based on the findings of the present study, it can be suggested that the positive effects of LLLT on collagen metabolism, collagen fiber deposition, and fiber organization may have provided better support to new muscle fiber formation in the treated groups in the last period. Further studies are necessary to elucidate the issue, as well as specify the type of collagen. 

Cox-2 immunoexpression was also evaluated. Cyclooxygenase is a key enzyme in the conversion of arachidonic acid into prostanoids. The expression of isoform cyclo-oxygenase-2 is relevant to many pathological processes, including inflammation, tissue repair, and ultimately carcinogenesis^18^. Our results showed a lower expression of Cox-2 in the group treated at 10 J/cm^2^ in the first period when compared to other groups. Albertini et al.^19^ also found that LLLT reduced the expression of Cox-2 messenger RNA (mRNA) in the subplantar muscle of rat paws subjected to carrageenan-induced inflammation. Furthermore, a previous study conducted by our research group found evidence that LLLT was able to promote a down-regulation of Cox-2 after induced muscle injury using LLLT at 50 J/cm2 for 6 sessions over 13 days^3^. We assumed that the down-regulation of Cox-2 is important because it attenuates the inflammatory process, inducing tissue repair in a short time period. 

One crucial point that needs to be determined in the field of laser therapy is the set of parameters required for optimal stimulation of tissue repair within the clinical setting. The complexity of the parameters involved in LLLT (i.e. fluency, power, pulse or continuous wave mode, and polarization state) has meant that a number of negative studies of LLLT as well as many positive studies have been published^20^^,^ 21. Meanwhile, the optimal dose of light for any particular application has to be evaluated, because lower or higher doses than this optimum value will result in negative out comes. These claims highlight the importance of studies exploring the effects of different wavelengths and energy densities on different tissues, in order to try to establish the safety of laser therapy and treatment parameters required for optimal stimulation^22^. It can be inferred from the present study that similar results in tissue healing were found by both fluencies employed. 

In summary, this study shows that LLLT had positive effects on muscle repair in rats, mainly with high fluency at 50 J/cm^2^. Although further studies and clinical trials are required, the findings of this study point to a promising use of this therapeutic modality for tissue repair. 
